# {*N*,*N*′-Bis[1-(2-pyrid­yl)ethyl­idene]propane-1,2-diamine}­bis(thio­cyanato-κ*N*)­nickel(II)

**DOI:** 10.1107/S1600536810029454

**Published:** 2010-07-31

**Authors:** Ning Wang

**Affiliations:** a Department of Chemical Engineering, Henan University of Urban Construction, Pingdingshan 467044, People’s Republic of China

## Abstract

In the title complex, [Ni(NCS)_2_(C_17_H_20_N_4_)], the Ni^2+^ ion (site symmetry 2) is coordinated by the *N*,*N*,*N*,*N*-tetra­dentate Schiff base ligand and two thio­cyanate ligands, forming a distorted NiN_6_ octa­hedral geometry, with the thio­cyanate N atoms in a *trans* orientation. The pendant methyl group of the central propane-1,2-diamine fragment of the ligand is statistically disordered over two sets of positions. In the crystal, weak aromatic π–π stacking between pyridine rings [centroid–centroid separation = 3.7081 (17) Å] may help to establish the packing.

## Related literature

For background to bis-Schiff bases in coordination chemistry, see: Yin *et al.* (1999 ▶); Costes *et al.* (2002 ▶); Haikarainen *et al.* (2001 ▶); Miyasaka *et al.* (2002 ▶); Ryaza­nov *et al.* (2002 ▶). For nickel complexes with Schiff bases, see: Liu *et al.* (2006 ▶); Li & Wang (2007 ▶); Liu *et al.* (2007 ▶); Ali *et al.* (2006 ▶); Knight *et al.* (2007 ▶).
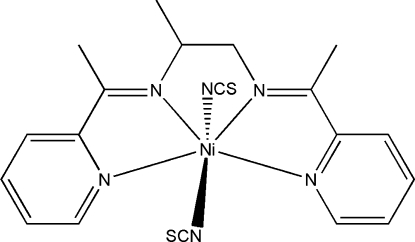


## Experimental

### 

#### Crystal data

[Ni(NCS)_2_(C_17_H_20_N_4_)]
*M* *_r_* = 455.24
Monoclinic, 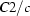

*a* = 12.431 (2) Å
*b* = 12.805 (2) Å
*c* = 13.613 (3) Å
β = 102.741 (2)°
*V* = 2113.5 (7) Å^3^
*Z* = 4
Mo *K*α radiation
μ = 1.13 mm^−1^
*T* = 298 K
0.27 × 0.25 × 0.23 mm

#### Data collection

Bruker APEXII CCD diffractometer
Absorption correction: multi-scan (*SADABS*; Sheldrick, 2004 ▶) *T*_min_ = 0.750, *T*_max_ = 0.781
8183 measured reflections
2311 independent reflections
1982 reflections with *I* > 2σ(*I*)
*R*_int_ = 0.023

#### Refinement

*R*[*F* ^2^ > 2σ(*F* ^2^)] = 0.037
*wR*(*F* ^2^) = 0.088
*S* = 1.09
2311 reflections
140 parameters
3 restraints
H atoms treated by a mixture of independent and constrained refinement
Δρ_max_ = 0.46 e Å^−3^
Δρ_min_ = −0.42 e Å^−3^

### 

Data collection: *APEX2* (Bruker, 2004 ▶); cell refinement: *SAINT* (Bruker, 2004 ▶); data reduction: *SAINT*; program(s) used to solve structure: *SHELXS97* (Sheldrick, 2008 ▶); program(s) used to refine structure: *SHELXL97* (Sheldrick, 2008 ▶); molecular graphics: *SHELXTL* (Sheldrick, 2008 ▶); software used to prepare material for publication: *SHELXTL*.

## Supplementary Material

Crystal structure: contains datablocks global, I. DOI: 10.1107/S1600536810029454/hb5571sup1.cif

Structure factors: contains datablocks I. DOI: 10.1107/S1600536810029454/hb5571Isup2.hkl

Additional supplementary materials:  crystallographic information; 3D view; checkCIF report

## Figures and Tables

**Table:** 

Ni1—N2	2.0157 (19)
Ni1—N3	2.060 (2)
Ni1—N1	2.111 (2)

**Table:** 

N2—Ni1—N2^i^	81.62 (11)
N2—Ni1—N3^i^	89.07 (9)
N2—Ni1—N3	95.39 (9)
N2—Ni1—N1^i^	158.89 (8)
N3—Ni1—N1^i^	91.23 (8)
N2—Ni1—N1	78.45 (8)
N3—Ni1—N1	85.93 (8)

Symmetry code: (i) 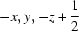.

**Fig. 1.**
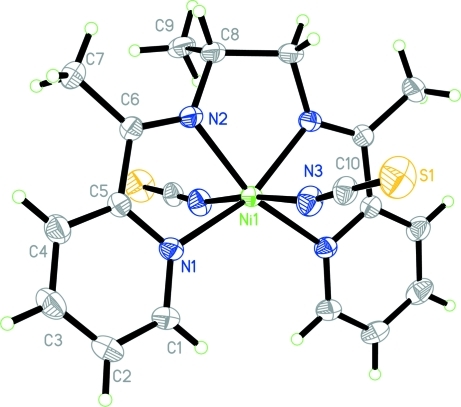
The molecular structure of the title compound, showing displacement ellipsoids drawn at the 30% probability level. Unlabeled atoms are related to labeled atoms by the symmetry operation - x, y, 1/2 - z. Only one orientation of C9 and its attached H atoms is shown.

## supplementary crystallographic information

### Comment

The bis-Schiff bases formed from aldehydes with diamines have been widely investigated in coordination chemistry (Yin *et al.*, 1999; Costes *et al.*, 2002; Haikarainen *et al.*, 2001; Miyasaka *et al.*, 2002; Ryazanov *et al.*, 2002). The complexes with such Schiff bases have proved to be of significant interest in the areas of catalysis, magnetism, medicinal and material chemistry. Although there have been numerous studies on the preparation and crystal structures of such complexes, the complexes with the Schiff base ligand *N*',*N''*-Bis(1-pyridin-2- ylethylidene)propane-1,2-diamine have never been reported. In the present paper, the title nickel(II) complex with the Schiff base ligand and thiocyanate is reported.

The molecule of the title complex, Fig. 1, possesses a crystallographic twofold rotation axis symmetry. The Ni atom is coordinated by four N atoms of a Schiff base ligand and two N atoms from two thiocyanate ligands, forming an octahedral geometry. The bond lengths (Table 1) related to the central Ni atom are comparable to those observed in other nickel(II) complexes with Schiff bases (Liu *et al.*, 2006; Li & Wang, 2007; Liu *et al.*, 2007; Ali *et al.*, 2006; Knight *et al.*, 2007).

### Experimental

To an ethanolic solution (30 ml) of 1,2-diaminopropane (0.074 g, 1 mmol) was added an ethanolic solution (30 ml) of 2-acetylpyridine (0.242 g, 2 mmol). The mixture was stirred at room temperature for 30 minutes. Then a solution of ammonium thiocyanate (0.152 g, 2 mmol) and nickel(II) nitrate hexahydrate (0.291 g, 1 mmol) in a minimum amount of ethanol was added, and the final mixture was further stirred at room temperature for 1 h. The clear solution was set aside for a week, yielding green blocks of (I).

### Refinement

The H8A and H8B atoms attached to C8 in the complex were located in a difference Fourier map and were refined with distance restraints of C–H = 0.97 (1) Å, and H···H = 1.55 (2) Å. All other H atoms were positioned geometrically and were constrained as riding atoms, with C–H distances of 0.93–0.96 Å, and *U*_iso_(H) set to 1.2 or 1.5*U*_eq_(C) of the parent atom. Rotating group models were used for the methyl groups.

### Crystal data

**Table:**

[Ni(NCS)_2_(C_17_H_20_N_4_)]	*F*(000) = 944
*M**_r_* = 455.24	*D*_x_ = 1.431 Mg m^−^^3^
Monoclinic, *C*2/*c*	Mo *K*α radiation, λ = 0.71073 Å
*a* = 12.431 (2) Å	Cell parameters from 2917 reflections
*b* = 12.805 (2) Å	θ = 2.3–25.0°
*c* = 13.613 (3) Å	µ = 1.13 mm^−^^1^
β = 102.741 (2)°	*T* = 298 K
*V* = 2113.5 (7) Å^3^	Block, green
*Z* = 4	0.27 × 0.25 × 0.23 mm

### Data collection

**Table:**

Bruker APEXII CCD diffractometer	2311 independent reflections
Radiation source: fine-focus sealed tube	1982 reflections with *I* > 2σ(*I*)
graphite	*R*_int_ = 0.023
ω scans	θ_max_ = 27.0°, θ_min_ = 2.3°
Absorption correction: multi-scan (*SADABS*; Sheldrick, 2004)	*h* = −15→15
*T*_min_ = 0.750, *T*_max_ = 0.781	*k* = −16→16
8183 measured reflections	*l* = −17→17

### Refinement

**Table:**

Refinement on *F*^2^	Primary atom site location: structure-invariant direct methods
Least-squares matrix: full	Secondary atom site location: difference Fourier map
*R*[*F*^2^ > 2σ(*F*^2^)] = 0.037	Hydrogen site location: inferred from neighbouring sites
*wR*(*F*^2^) = 0.088	H atoms treated by a mixture of independent and constrained refinement
*S* = 1.09	*w* = 1/[σ^2^(*F*_o_^2^) + (0.0271*P*)^2^ + 3.1898*P*] where *P* = (*F*_o_^2^ + 2*F*_c_^2^)/3
2311 reflections	(Δ/σ)_max_ < 0.001
140 parameters	Δρ_max_ = 0.46 e Å^−^^3^
3 restraints	Δρ_min_ = −0.42 e Å^−^^3^

### Special details

**Table:**

Geometry. All e.s.d.'s (except the e.s.d. in the dihedral angle between two l.s. planes) are estimated using the full covariance matrix. The cell e.s.d.'s are taken into account individually in the estimation of e.s.d.'s in distances, angles and torsion angles; correlations between e.s.d.'s in cell parameters are only used when they are defined by crystal symmetry. An approximate (isotropic) treatment of cell e.s.d.'s is used for estimating e.s.d.'s involving l.s. planes.
Refinement. Refinement of *F*^2^ against ALL reflections. The weighted *R*-factor *wR* and goodness of fit *S* are based on *F*^2^, conventional *R*-factors *R* are based on *F*, with *F* set to zero for negative *F*^2^. The threshold expression of *F*^2^ > σ(*F*^2^) is used only for calculating *R*-factors(gt) *etc*. and is not relevant to the choice of reflections for refinement. *R*-factors based on *F*^2^ are statistically about twice as large as those based on *F*, and *R*- factors based on ALL data will be even larger.

### Fractional atomic coordinates and isotropic or equivalent isotropic displacement parameters (Å^2^)

**Table:**

	*x*	*y*	*z*	*U*_iso_*/*U*_eq_	Occ. (<1)
Ni1	0.0000	0.18939 (3)	0.2500	0.03735 (14)	
S1	0.36209 (7)	0.21125 (8)	0.18060 (8)	0.0830 (3)	
N1	0.06978 (16)	0.10959 (15)	0.38468 (15)	0.0409 (5)	
N2	0.03606 (17)	0.30853 (15)	0.34856 (15)	0.0428 (5)	
N3	0.15474 (19)	0.18115 (19)	0.21915 (17)	0.0552 (6)	
C1	0.0951 (2)	0.0091 (2)	0.3988 (2)	0.0497 (6)	
H1	0.0766	−0.0361	0.3442	0.060*	
C2	0.1477 (2)	−0.0311 (2)	0.4910 (2)	0.0593 (7)	
H2	0.1657	−0.1016	0.4980	0.071*	
C3	0.1726 (3)	0.0348 (2)	0.5717 (2)	0.0652 (8)	
H3	0.2066	0.0094	0.6349	0.078*	
C4	0.1469 (2)	0.1393 (2)	0.5586 (2)	0.0568 (7)	
H4	0.1631	0.1851	0.6129	0.068*	
C5	0.0970 (2)	0.17503 (19)	0.46397 (18)	0.0419 (5)	
C6	0.0706 (2)	0.28796 (19)	0.44138 (18)	0.0432 (6)	
C7	0.0860 (3)	0.3646 (2)	0.5261 (2)	0.0633 (8)	
H7A	0.0774	0.4343	0.4995	0.095*	
H7B	0.1585	0.3568	0.5681	0.095*	
H7C	0.0318	0.3521	0.5653	0.095*	
C8	−0.0029 (3)	0.41034 (19)	0.3058 (2)	0.0511 (6)	
C9	−0.1228 (5)	0.4304 (5)	0.3185 (5)	0.0575 (15)	0.50
H9A	−0.1718	0.3803	0.2794	0.086*	0.50
H9B	−0.1453	0.4995	0.2957	0.086*	0.50
H9C	−0.1252	0.4236	0.3882	0.086*	0.50
C10	0.2400 (2)	0.1951 (2)	0.20181 (18)	0.0475 (6)	
H8A	0.0431 (18)	0.4673 (15)	0.3394 (18)	0.057*	
H8B	−0.0770 (17)	0.425 (4)	0.313 (6)	0.057*	0.50

### Atomic displacement parameters (Å^2^)

**Table:**

	*U*^11^	*U*^22^	*U*^33^	*U*^12^	*U*^13^	*U*^23^
Ni1	0.0420 (3)	0.0318 (2)	0.0380 (2)	0.000	0.00829 (18)	0.000
S1	0.0502 (5)	0.1068 (8)	0.0979 (7)	−0.0146 (4)	0.0290 (5)	−0.0109 (5)
N1	0.0403 (11)	0.0382 (11)	0.0443 (11)	0.0025 (8)	0.0097 (9)	0.0035 (9)
N2	0.0512 (12)	0.0345 (10)	0.0419 (11)	0.0027 (9)	0.0086 (9)	−0.0015 (9)
N3	0.0495 (13)	0.0646 (15)	0.0532 (13)	−0.0075 (11)	0.0152 (11)	−0.0068 (11)
C1	0.0534 (15)	0.0383 (13)	0.0572 (16)	0.0031 (11)	0.0120 (12)	0.0031 (11)
C2	0.0607 (18)	0.0464 (15)	0.0686 (19)	0.0037 (13)	0.0094 (15)	0.0175 (14)
C3	0.070 (2)	0.0642 (19)	0.0554 (17)	−0.0016 (15)	0.0001 (15)	0.0204 (15)
C4	0.0646 (18)	0.0579 (17)	0.0450 (15)	−0.0044 (14)	0.0060 (13)	0.0068 (13)
C5	0.0413 (13)	0.0437 (13)	0.0412 (13)	−0.0012 (10)	0.0104 (10)	0.0029 (10)
C6	0.0464 (14)	0.0425 (13)	0.0418 (13)	−0.0003 (10)	0.0121 (11)	−0.0029 (10)
C7	0.091 (2)	0.0538 (17)	0.0455 (15)	0.0042 (16)	0.0152 (15)	−0.0087 (13)
C8	0.0694 (18)	0.0326 (12)	0.0490 (15)	0.0042 (12)	0.0083 (14)	−0.0008 (11)
C9	0.069 (4)	0.038 (3)	0.059 (3)	0.007 (3)	0.001 (3)	−0.008 (2)
C10	0.0474 (15)	0.0542 (15)	0.0393 (13)	−0.0041 (12)	0.0063 (11)	−0.0061 (11)

### Geometric parameters (Å, °)

**Table:**

Ni1—N2	2.0157 (19)	C3—H3	0.9300
Ni1—N2^i^	2.0157 (19)	C4—C5	1.379 (3)
Ni1—N3^i^	2.060 (2)	C4—H4	0.9300
Ni1—N3	2.060 (2)	C5—C6	1.499 (3)
Ni1—N1^i^	2.111 (2)	C6—C7	1.495 (3)
Ni1—N1	2.111 (2)	C7—H7A	0.9600
S1—C10	1.620 (3)	C7—H7B	0.9600
N1—C1	1.329 (3)	C7—H7C	0.9600
N1—C5	1.349 (3)	C8—C8^i^	1.536 (5)
N2—C6	1.269 (3)	C8—C9	1.560 (6)
N2—C8	1.466 (3)	C8—H8A	0.976 (10)
N3—C10	1.149 (3)	C8—H8B	0.967 (10)
C1—C2	1.380 (4)	C9—H9A	0.9600
C1—H1	0.9300	C9—H9B	0.9600
C2—C3	1.365 (4)	C9—H9C	0.9600
C2—H2	0.9300	C9—H8B	0.594 (13)
C3—C4	1.378 (4)		
			
N2—Ni1—N2^i^	81.62 (11)	N1—C5—C4	121.5 (2)
N2—Ni1—N3^i^	89.07 (9)	N1—C5—C6	115.5 (2)
N2^i^—Ni1—N3^i^	95.39 (9)	C4—C5—C6	123.0 (2)
N2—Ni1—N3	95.39 (9)	N2—C6—C7	126.1 (2)
N2^i^—Ni1—N3	89.07 (9)	N2—C6—C5	114.5 (2)
N3^i^—Ni1—N3	174.13 (14)	C7—C6—C5	119.4 (2)
N2—Ni1—N1^i^	158.89 (8)	C6—C7—H7A	109.5
N2^i^—Ni1—N1^i^	78.45 (8)	C6—C7—H7B	109.5
N3^i^—Ni1—N1^i^	85.93 (9)	H7A—C7—H7B	109.5
N3—Ni1—N1^i^	91.23 (8)	C6—C7—H7C	109.5
N2—Ni1—N1	78.45 (8)	H7A—C7—H7C	109.5
N2^i^—Ni1—N1	158.89 (8)	H7B—C7—H7C	109.5
N3^i^—Ni1—N1	91.23 (8)	N2—C8—C8^i^	108.03 (17)
N3—Ni1—N1	85.93 (8)	N2—C8—C9	110.0 (3)
N1^i^—Ni1—N1	122.10 (11)	C8^i^—C8—C9	111.3 (4)
C1—N1—C5	118.5 (2)	N2—C8—H8A	111.8 (16)
C1—N1—Ni1	129.29 (18)	C8^i^—C8—H8A	108.2 (16)
C5—N1—Ni1	112.12 (15)	C9—C8—H8A	107.5 (16)
C6—N2—C8	126.1 (2)	N2—C8—H8B	112 (4)
C6—N2—Ni1	118.83 (17)	C8^i^—C8—H8B	111 (5)
C8—N2—Ni1	113.78 (15)	C9—C8—H8B	2(4)
C10—N3—Ni1	168.1 (2)	H8A—C8—H8B	106 (2)
N1—C1—C2	122.8 (3)	C8—C9—H9A	109.5
N1—C1—H1	118.6	C8—C9—H9B	109.5
C2—C1—H1	118.6	H9A—C9—H9B	109.5
C3—C2—C1	118.6 (3)	C8—C9—H9C	109.5
C3—C2—H2	120.7	H9A—C9—H9C	109.5
C1—C2—H2	120.7	H9B—C9—H9C	109.5
C2—C3—C4	119.4 (3)	C8—C9—H8B	3(7)
C2—C3—H3	120.3	H9A—C9—H8B	111.4
C4—C3—H3	120.3	H9B—C9—H8B	106.2
C3—C4—C5	119.2 (3)	H9C—C9—H8B	110.7
C3—C4—H4	120.4	N3—C10—S1	177.8 (3)
C5—C4—H4	120.4		

Symmetry codes: (i) −*x*, *y*, −*z*+1/2.
